# Correction: Optimization and characterization of miRNA-129-5p-encapsulated poly (lactic-*co*-glycolic acid) nanoparticles to reprogram activated microglia

**DOI:** 10.1039/d3na90065g

**Published:** 2023-07-04

**Authors:** Irina Kalashnikova, Heather R. Campbell, Daniel Kolpek, Jonghyuck Park

**Affiliations:** a Department of Pharmaceutical Sciences, College of Pharmacy, University of Kentucky 789 S. Limestone Lexington KY 40506 USA jonghyuck.park@uky.edu +1-859-257-1850; b Spinal Cord and Brain Injury Research Center, College of Medicine, University of Kentucky Lexington KY USA

## Abstract

Correction for ‘Optimization and characterization of miRNA-129-5p-encapsulated poly (lactic-*co*-glycolic acid) nanoparticles to reprogram activated microglia’ by Irina Kalashnikova, *et al.*, *Nanoscale Adv.*, 2023, https://doi.org/10.1039/D3NA00149K.

The authors regret that the name of the author Heather R. Campbell was incorrectly spelt in the original manuscript. The correct spelling is listed above.

The Royal Society of Chemistry apologises for these errors and any consequent inconvenience to authors and readers.

## Supplementary Material

